# Formulation of Vermicelli Mixed Corn and Rice Flour with Additional Carrageenan and Its Economic Value

**DOI:** 10.1155/2022/7387223

**Published:** 2022-11-16

**Authors:** Moh Saeri, Joko Mariyono, Evy Latifah, Aniswatul Khamidah, Ita Yustina, Sugiono Sugiono, Khojin Supriadi, Herman Subagio, Sri Satya Antarlina

**Affiliations:** ^1^Research Center for Behavioral and Circular Economics, Research Center for Behavioral and Circular Economics, Research Organization for Governance Economy and Community Welfare, National Research and Innovation Agency (BRIN), Indonesia; ^2^Agribusiness Study Program, Faculty of Animal and Agricultural Sciences, Universitas Diponegoro, Indonesia; ^3^Research Center for Horticulture and Plantation, Research Organization for Agriculture and Food, National Research and Innovation Agency (BRIN), Indonesia; ^4^Research Center for Food Technology and Processing, Research Organization for Agriculture and Food, National Research and Innovation Agency (BRIN), Indonesia; ^5^Research Center for Food Crops, Research Organization for Agriculture and Food, National Research and Innovation Agency (BRIN), Indonesia; ^6^Research Center for Macroeconomics and Finance, Research Organization for Governance Economy and Community Welfare, National Research and Innovation Agency (BRIN), Indonesia

## Abstract

The study was aimed at obtaining a vermicelli formulation on a mixture of corn and rice flour, adding carrageenan and its economic analysis. The experiment applied a 2-factorial randomized block design, where factor 1 was a mixture of corn and rice flour (5 levels) and factor 2 was carrageenan concentration (5 levels), repeated three times. The data were analyzed using ANOVA provided in SPSS. When there were significant differences, the analysis proceeded with DMRT at a level of 5% to see differences among treatments. The results show that the higher the corn flour and carrageenan concentration, the higher the vermicelli's ash and fat content. The formulation produces wet vermicelli with a good appearance. The production of vermicelli uses an extruder method. The selected vermicelli formulation was a mixture of 25% corn flour with 75% rice flour and the addition of 0.6% carrageenan. The characteristics of the wet vermicelli are moisture content of 42.84%, ash content of 0.21% on a wet basis (wb), and fat content of 0.43% wb. The organoleptic test of vermicelli was color 3.9 (liked), aroma 3.6 (liked), texture 2.6 (quite soft), taste 3.7 (liked), and general appearance 3.5 (liked). Economically, making vermicelli made of corn and rice flour is profitable because the R/C ratio value is greater than one, which is 2.27. The resulting wet vermicelli resembles wet noodles, large in size and yellow in color, so it can be recommended as gluten-free noodles, suitable for consumption by people with gluten allergies.

## 1. Introduction

Agriculture and food sectors currently contribute to the economies all over the world. The contribution is more significant in agrarian countries, including Indonesia, than its counterparts since the agricultural products become raw material in the value chains. In Indonesia, the agriculture and food system industry contributes half of the total employment and one-third of the country's national income [1].

Rice flour is one of the raw materials for making vermicelli, an important food type. However, using rice as a raw material might affect the national rice self-sufficiency program. Thus, vermicelli production needs alternative materials to substitute rice, so the production does not influence the program. One of the potential substitutes is corn flour.

However, the vermicelli made of corn has an unexpected texture, which might be unfavorable to the consumers. In order to get a firm and soft texture of corn vermicelli, a texture-forming, namely, carrageenan material, is added to the production [2, 3]. Carrageenan is a group of galactose polysaccharides extracted from seaweed. Carrageenan can form a thermoreversible gel or viscous solution when added to a salt solution, so it is widely used as a gelling agent, thickener, and stabilizer in various industries such as food [4, 5].

Vermicelli is consumed as a source of carbohydrates as a staple food to either complement or substitute rice. Vermicelli is a type of noodle made of rice flour. In order to produce vermicelli with good quality, raw materials with specific starch characteristics are needed. The ideal starch for vermicelli raw materials is starch that has a small granule size, high amylose content, degree of swelling, and limited solubility and the characteristics of type C paste (it does not have a peak viscosity, but the viscosity tends to be high and does not decrease during the heating and stirring process) [6].

Corn flour has properties similar to the special rice flour used as vermicelli ingredients; it has a high amylose content and is flavorful, making it suitable for making vermicelli. According to [7], local varieties of yellow corn starch contain an amylose content of 59.83%, while [8] reported that corn starch contained 37.10-57.29% amylose. According to [9, 10], starch with high amylose content is ideal for making *starch noodles or instant starch noodles*.

Corn flour can be used as an alternative material in the manufacture of vermicelli because it has several advantages. According to [11, 12], corn has a higher protein content (9.5%) than rice (7.4%). In addition, the mineral and vitamin content between rice and corn is also almost the same. The advantage of corn compared to other types of cereals is the yellow color of corn. The yellow color of corn is due to the carotenoid content. Yellow corn contains carotenoids ranging from 6.4 to 11.3 g/g, 22% beta-carotene, and 51% xanthophylls. The main xanthophyll pigments are lutein and zeaxanthin [13, 14].

Corn has a low glycemic index (GI) of 46, and the index is lower than rice, which accounts for 72. It is considered a high GI since the value is above 70, moderate if it is between 56 to 69, and low below 55 [15]. Foods with a high GI will cause a sudden spike in blood sugar levels. Blood sugar levels become unstable, and the body suddenly feels full but quickly becomes hungry again, so consumption of corn is recommended for people with diabetes.

Farmers have developed varieties of corn and rice in the market, so it is necessary to characterize the seeds to produce flour as raw material for vermicelli from various varieties, especially those that produce high yields. The government continues to promote food diversification programs considering the high demand for rice and other imported raw materials. The law mandates the government to build solid food security by focusing on increasing national food production capacity for five strategic food commodities: rice, corn, soybean, sugar cane, and beef. Corn is one of the local commodities to support Indonesian food security. Corn contains nutritious values and has been considered a staple food in some areas of Indonesia since it fulfills national food needs of 26.0% (19,612,435 t/year) [16]. From an economic point of view, the price of corn is about 44.6% lower than that of rice [16]. Using a substitute of corn material for making vermicelli from corn is expected to increase business income. The study was aimed at obtaining wet vermicelli formulations on various compositions of corn and rice flour mixture, with the addition of carrageenan and the economic efficiency value of the formula.

## 2. Materials and Methods

### 2.1. Place and Material

The research was conducted at the Postharvest Laboratory of Assessment Institute for Agricultural Technology (AIAT) East Java in Malang and the Food Laboratory of the Center for Agricultural Postharvest Research and Development in Bogor, Indonesia. The main ingredients were corn seeds and rice. Corn varieties are DK 88, Bisi 18, DK 979, DK 99, and Pertiwi, and corn is obtained from farmers' plantations in Kediri Regency, East Java. Rice varieties are Ciherang, Inpari 19, Inpari 4, Inpari 30, and IR 64, obtained from farmers' plantations in Malang Regency, East Java. Type and supporting materials were tapioca flour, carrageenan, and chemicals for analysis.

### 2.2. Analysis Design and Procedure

The treatment of the vermicelli formulation was a mixture of corn flour with rice flour and carrageenan (as a stabilizer, thickener, texturizer, and gel). There were 5 levels of corn flour: rice flour formulations, namely, (1) 0 : 100 (control); (2) 25 : 75; (3) 50 : 50; (4) 75 : 25; and (5) 100 : 0. There were 5 levels of added carrageenan, namely, 0, 0.3, 0.6, 0.9, and 1.2% *w*/*w* [6]. In factorial randomized experimental design (2 factors), factor 1 was a mixture of corn and rice flour (5 levels), and factor 2 was carrageenan concentration (5 levels), repeated three times. The combination of treatments was 75 samples. This study analyzed data using analysis of variances (ANOVA) to test the significant difference among the treatments. If the ANOVA shows significance at 5% level, the test proceeds with the smallest significant difference test at 5% level.

### 2.3. Vermicelli Characteristic Analysis

The yield of flour is calculated based on the percentage of flour weight (yield) divided by the weight of seeds (initial) used as flour and then multiplied by one hundred percent [17]. The chemical analysis of corn vermicelli includes the moisture content of the oven method. The ash content using the method of ashing the sample is carried out using a muffle furnace at a temperature of 550°C—the fat content using the Soxhlet method [18, 19]. Organoleptic tests on vermicelli after steaming (15 minutes) using the hedonic method were carried out on color, aroma (smell), texture, taste, and general acceptance, using 30 semitrained panelists. The test is carried out based on a hedonic scale score, and the results are expressed in numbers on color, aroma, taste, and general acceptance, namely, 1 = very dislike, 2 = dislike, 3 = quite, 4 = like, and 5 = really like it, and regarding texture, namely, 1 = very soft, 2 = soft, 3 = moderate, 4 = hard, and 5 = very hard [20].

### 2.4. Financial Analysis of Vermicelli Formula of Corn Flour and Rice Flour

Financial analysis is an essential part of product innovation since the analysis will determine the feasibility of the innovation. The community will adopt the innovation when the production is profitable [21]. This analysis was conducted to determine the economic performance of producing the vermicelli formula. The performance is measured using cost, revenue, profit, and R/C ratio [22].

The cost is formulated as TC = TVC + TFC, where TC is defined as the total cost, TVC is the total variable cost, and TFC is the total fixed cost. The costs include the monetary value of inputs spent to finance the vermicelli products. Revenue or total revenue (TR) is the total money received from products that are successfully sold or the multiplication of the number of products produced (Q) with the selling price per unit product (P), which is formulated as TR = P × Q. Profit (*Π*) is the difference between total revenue and total cost, with the following equation: *Π* = TR–TC. Then, the R/C ratio compares revenue and total cost, formulated as R/C ratio = TR/TC.

The criteria used to assess the R/C ratio are as follows:

- R/C ratio > 1 means that the business of processing vermicelli products is profitable
- R/C ratio < 1 means that the business of processing vermicelli products is detrimental
- R/C ratio = 1 means that the business of processing vermicelli products has a return on investment (break-even point/BEP).

### 2.5. Corn Flour Making Method

Corn seeds were coarsely ground into corn kernels. Corn kernels are cleaned from the husk and organs. After cleaning, the corn kernels are soaked for about 3 hours and then drained. In moist conditions, corn kernels were ground into flour. The corn flour was dried in an oven at a temperature of 60°C for 16 minutes until the moisture content was 12% and then manually sieved with a particle size of flour that passed a 100 mesh sieve.

### 2.6. Rice Flour Making Method

The rice is washed and soaked for about 3 hours and then drained. In moist conditions, the rice was ground into flour. The flour was dried in an oven at 60°C for 16 minutes until the moisture content was 12% and then manually sieved with a particle size of flour that passed a 100 mesh sieve.

### 2.7. Vermicelli Making Method

**Figure 1:**
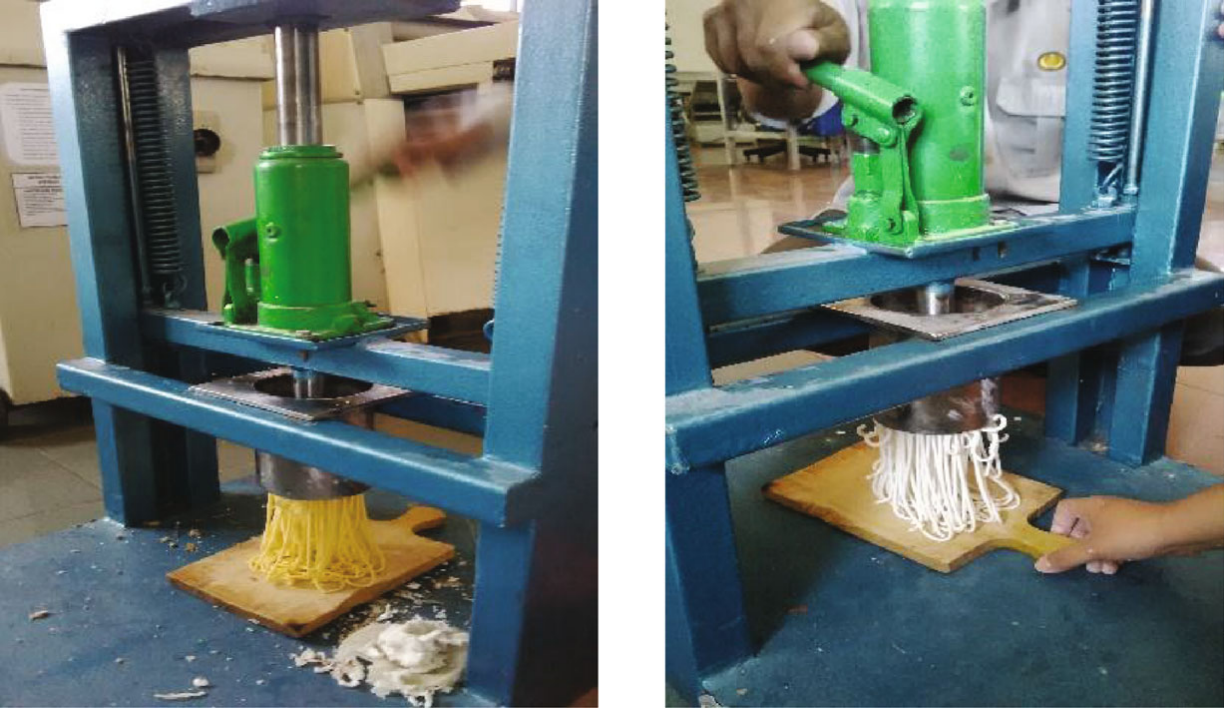
Corn flour vermicelli (left) and rice flour vermicelli (right) and a hydraulic press system for wet vermicelli presses.

**Figure 2:**
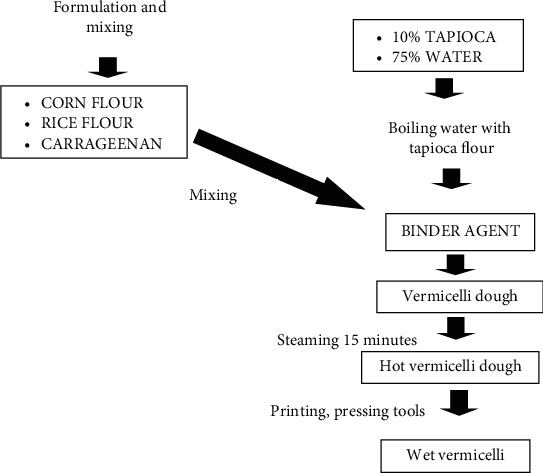
Flowchart of making wet vermicelli.

After the treatment, mix rice flour, corn flour, and carrageenan. According to [6], the binding of particles in the manufacture of corn vermicelli uses a hydrocolloid binder (binder agent). Making a binder agent by adding 10% tapioca flour and 75% water, the method is boiling tapioca flour with water until it thickens to form a gel. Then, enter the mixture of corn flour, rice flour, and carrageenan, stir until it forms a soft dough, and then steam for 15 minutes. In hot conditions, the dough is molded by pressing it into vermicelli using a tool with hydraulic power pressure (Figure 1). The flow chart for making wet vermicelli is presented in Figure 2. The ingredients for making vermicelli are corn flour of DK 99 variety and rice flour of Inpari 30 variety. Vermicelli making is made in one batch. For chemical analysis, samples of vermicelli were taken for each treatment as much as 20 g. The organoleptic test sample of 5 g for each treatment was served to one panelist.

### 2.8. Equipment

The equipment used was a milling tool to form kernels of corn seeds and a flouring tool to produce corn flour and rice flour. The sifter is used to sieve corn flour and rice flour to obtain a flour size of 100 mesh. Extruder is used for printing vermicelli. Soaking container is a tool used to soak corn and rice seeds. Stoves and pans were used to steam the dough until cooked. Equipment is used to analyze raw materials and vermicelli's physical and chemical properties—complementary equipment for organoleptic tests.

## 3. Result and Discussion

### 3.1. Physical Characteristics of Corn Seed and Rice Grain

The physical characteristics of corn seed and rice grain of five varieties are presented in Table 1. There were differences in the physical characteristics of the five varieties of corn seed and rice grain (*p* < 0.01). Bisi 18, DK 99, and Pertiwi varieties of corn had the same seed length, while DK 88 and DK 979 were shorter than the others. The varieties of corn include Bisi 18 (11.43 mm), DK 99 (11.07 mm), and Pertiwi (11.72 mm). They had similar length, width, and weight characteristics as 100 seeds. Likewise, DK 88 (9.95 mm) and DK 979 (9.90 mm) had a length, width, and weight lower than 100 seeds. The thickness of the seeds has a different pattern, and DK 979 variety was the thickest (0.464 cm), while DK 88 (0.385 cm) was the thinnest. Research results by [23] showed that corn has a length of seeds ranging from 7.98 to 10.06 mm, a width of 2.84-5.87 mm, and a height of 7.89-10.51 mm. The highest weight of 100 corn seeds was the Pertiwi variety (34.044 g), and the lowest was the DK 99 variety (29.493 g). The Pertiwi variety showed the largest seed size, and the DK 99 variety showed the smallest seed size of the five varieties observed.

For the physical characteristics of the rice grain of five varieties, it appears that the longest rice grain was IR 64 (7.1 mm), while Inpari 19 was the shortest (6.4 mm). Rice grain width almost did not differ between varieties, the lowest being Inpari 4 (1.9 mm). The length variation of the five rice grain varieties was 6.4-7.1 mm, and the width was 1.9-2.2 mm. There was no correlation between the length and width of rice grain and weight. The research results by [24] showed that ten varieties of long rice grain varied between 5.5 and 8.2 mm and width 1.9-2.8 mm. Rice grains were classified based on the length of a rice grain into several groups, namely, short (<5.51 mm), medium (5.51-6.60 mm), long (6.61-7.50 mm), and very long (>7.50 mm) [25]. The five varieties observed were classified as long rice grain based on this classification.

The shape grain of rice can be determined based on the ratio between the length and width of the rice grain, namely, bold (<2.1), medium (>2.1-3), and slender (>3) [24]. The measurement results of the ratio of length and width of rice grain were 3.05-3.63, so the rice grain of all varieties was categorized as lean. The highest weight of 100 rice grains was IR 64 (2.02 g), while the lowest were Inpari 19 and Inpari 4, which were 1.86 g and 1.88 g, respectively. The morphological characteristics of rice grains were different for each rice variety. Rice grain length was a distinctive character that can be used to observe differences in rice varieties and the shape, width, and thickness of rice grain.

### 3.2. Corn and Rice Yield of Flour

**Figure 3:**
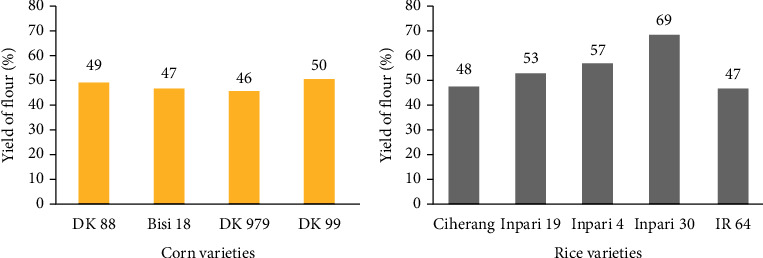
Yield of corn and rice flour.

Varieties of corn and rice affect the yield of flour produced (*p* < 0.01) (Figure 3). The yield of flour produced varied among the five varieties observed. For the yield of corn flour at the size of the 100 mesh sieve, the highest was the DK 99 variety (50.42%), and the lowest was the Pertiwi variety (41.20%). For the yield of rice flour at the size of the 100 mesh sieve, the Inpari 30 variety (68.83%) produced the highest yield of flour, while the lowest was Ciherang (47.5%). The yield of flour was the ratio of the weight of the resulting product to the weight of the raw materials used. The highest yield of flour is related to the monetary value. The higher the yield of flour, the higher the monetary value. Therefore, the varieties that produced the highest yield of flour in the production of vermicelli were selected, namely, DK 99 for corn varieties and Inpari 30 for rice varieties.

The yield of flour calculation is based on the dry weight of the material. The yield of flour states the efficiency value of the processing process so that it can be seen as the amount of flour produced from the initial raw material. The yield of corn flour obtained by research by [26] was 36% on an 80 mesh sieve and with the treatment of soaking the seeds in water for 20 hours. However, the corn fermentation treatment using *Lactobacillus casei* resulted in the highest yield (55.2%) at 60 hours of immersion. The presence of lactic acid bacteria (*L. bulgaricus* and *L. casei*) was able to degrade corn cell walls so that starch granules came out of the cells, which facilitated the milling process so that the yield increased.

**Figure 4:**
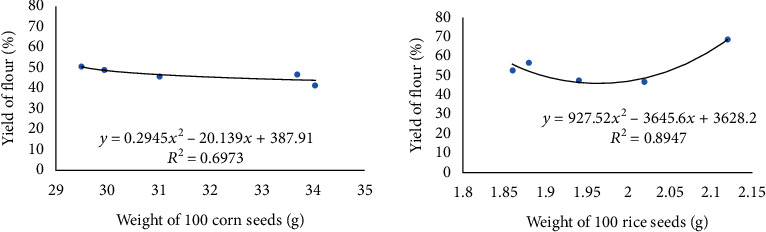
The relationship between flour yield and weight of 100 seeds.

Figure 4 shows the relationship between seed weight and flour yield. It appears that corn and rice have different patterns. There is a more significant relationship in the yield of rice flour which has a correlation coefficient value (*R*^2^ = 0.8947) with the quadratic function equation *y* = 927.52*x*^2^ − 3645.6*x* + 3628.2, compared to the yield of corn flour (*R*^2^ = 0.6973) with the quadratic function equation *y* = 0.2945*x*^2^ − 20.139*x* + 387.91. This shows the difference between rice and corn. The heavier the rice, the higher the flour yield, while the corn flour is different.

### 3.3. Yield of Corn and Rice Vermicelli

#### 3.3.1. The Yield of Wet Vermicelli from a Mixture of Corn and Rice Flour

**Figure 5:**
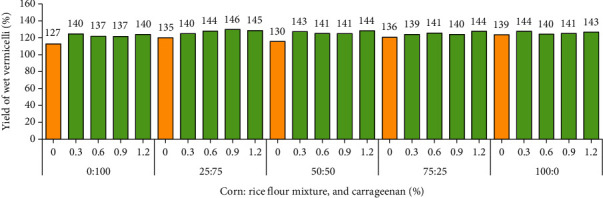
Yield of wet vermicelli (%) (average content of 40.96%). Note: 0 : 100 (0% corn: 100% rice); 25 : 75 (25% corn: 75% rice); 50 : 50 (50% corn: 50% rice); 75 : 25 (75% corn: 25% rice); and 100 : 0 (100% corn: 0% rice). Carrageenan: 0, 0.3, 0.6, 0.9, and 1.2.

The yield of wet vermicelli is presented in Figure 5. It appears that the higher the addition of corn flour, the higher the yield of vermicelli. Likewise, adding carrageenan at higher concentrations can increase the yield of vermicelli. This is because corn fiber content is relatively higher than rice fiber content; corn fiber content is 0.79–2.48% [19], and rice fiber content is 0.48–0.85% [27]. Fiber is a structure-forming material, and fiber components consist of lignin and cellulose so that in milling seeds, the fiber content can increase the yield of higher flour. Likewise, carrageenan is a polysaccharide group of galactose and can form a gel, causing an increase in fiber components [4, 5, 28].

#### 3.3.2. Vermicelli Chemical Content

Chemical analysis of vermicelli was carried out to determine the moisture, ash, and fat content. The results of the chemical analysis of vermicelli are presented in Table 2. The moisture, ash, and fat content of vermicelli are influenced by the interaction of two factors (*p* < 0.01), namely, flour composition and carrageenan concentration [29, 30]. The lowest moisture content of vermicelli was from the combination treatment of rice flour (100%) without the addition of carrageenan (0%), which was 30.695%, while the highest moisture content was from the combination of treatment with a mixture of 50% corn flour+50% rice flour with the addition of carrageenan at a concentration of 0.3%, which was 43.830%.

The moisture content of a food material significantly affects its shelf life because microbes are increasingly inhibited by the lower moisture content [31]. The higher the moisture content of the food, the faster the damage by microbial activity. This is because the microbes need free moisture for growth [32]. The vermicelli produced was wet, so the moisture content is high and cannot be stored at room temperature.

The lowest ash content of vermicelli was in the combination of 50% corn flour+50% rice flour mixed with the addition of carrageenan at a concentration of 0.3%, namely, 0.065% wet basis (wb), while the highest ash content was in the combination treatment of 75% corn flour+25% rice flour with the addition of carrageenan at a concentration of 1.2%, namely, 0.725%wb. The ash content in vermicelli was strongly influenced by the mineral content [33]. Carrageenan was made from seaweed. The addition of carrageenan at higher concentrations increased the ash content of the vermicelli. According to [34], carrageenan ash content is around 16.19-22.44%, and moisture content is 13.76-19.46%.

The lowest fat content of vermicelli was from the combination treatment of rice flour (100%) with the addition of carrageenan at a concentration of 0.9%, which was 0.325%wb, while the highest fat content was from the combination treatment of a mixture of 50% corn flour+50% rice flour with the addition of carrageenan at a concentration of 1.2%, which was 0.450%wb. The addition of corn flour increases the fat content of the vermicelli because the raw material corn had a higher fat content than rice. According to [35], the fat content of corn flour from the five varieties ranged from 1.62 to 1.85%, while from the research of [36], the fat content of rice flour was 0.35-0.39%.

#### 3.3.3. Vermicelli Organoleptic Test

**Figure 6:**
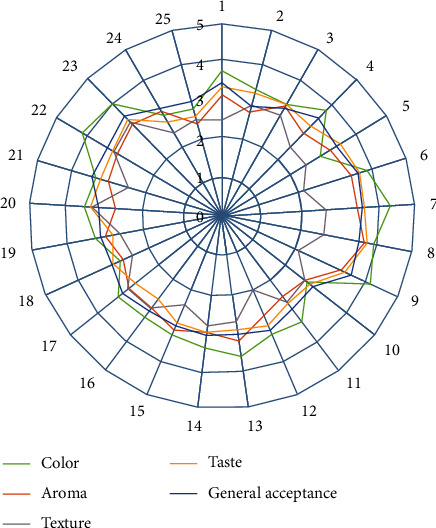
Results of organoleptic test of corn and rice mixture vermicelli (treatments 1 to 25 are presented in Table 3).

The organoleptic test of vermicelli was influenced by the interaction between two factors (*p* < 0.01), namely, the mixed flour formulation and carrageenan concentration. The results of the organoleptic test of vermicelli are presented in Table 3 and Figure 6. The most preferred color of vermicelli was in the treatment of a mixture of 25% corn flour and 75% rice flour, with the addition of carrageenan 0.3%, a score of 4.2 (like). The preferred color was corn flour mixed vermicelli because the color was brighter yellow when compared to white rice flour vermicelli. Corn flour contains yellow pigments (carotenoids) [37]. The assessment range for the color of the vermicelli was between 2.80 and 4.10, with the criteria being enough to like.

The most preferred aroma was a mixture formulation of 25% corn flour and 75% rice flour, adding 0.6% carrageenan, with a score of 3.9 (like). The aroma assessment range was between 2.45 and 3.55, with the criteria of dislike to moderate (neutral). The criteria for assessing the texture of the vermicelli were classified as very soft to very hard. The highest texture rating was from a mixed formulation of 100% corn flour, with 0.6% carrageenan, a score of 3.4 (moderate), while the vermicelli texture with the softest assessment was from a mixture formulation of 25% corn flour and 75% flour. Rice without adding carrageenan (0%) gets scored 2.1 (soft). It appears that the combination of a mixture of corn flour with the addition of carrageenan can increase the hardness of the vermicelli texture.

The most preferred vermicelli taste was a mixture formulation of 25% corn flour and 75% rice flour, with the addition of 0.6% carrageenan, a score of 3.7 (like). The lowest rating is with a score of 2.7 (dislike), in several formulations. The most favorable general acceptance is the formulation of a mixture of 25% corn flour and 75% rice flour, without the addition of carrageenan (0%), but not different from 100% corn flour, 0.6% carrageenan addition, with a score of 3.6 (like). The lowest rating has a score of 2.9 (moderate).

In addition, it was also influenced by the amylose and amylopectin levels of flour. The lower the amylose content in the material causes the gel structure to be weaker and causes the dissolved solids to be greater so that the vermicelli texture is low (soft). The texture of the vermicelli was also affected by the stickiness on the surface because the amylopectin molecules form amorphous or less compact regions, making it easier for water to penetrate [38]. The amylose content in corn flour was higher than in rice flour, so the vermicelli with a higher proportion of corn flour had a harder texture. Corn flour amylose content was 28.0% [26], while rice flour was 11.78% [39].

### 3.4. Economic Analysis of Vermicelli Production

Economic analysis of vermicelli production is a way to control finances so that the level of business success achieved during vermicelli production takes place. Producers use this analysis to make calculations and determine steps to improve and increase profits in their business activities [40].

Cost analysis was a tool used to explain the relationship between costs and the company's volume or the number of products. Cost analysis was formed based on two costs, namely, fixed costs and variable costs. Fixed costs were costs incurred in a fixed amount, not influenced by the volume to be produced, such as depreciation costs of tools and machines used. Variable costs were incurred according to the production volume, for example, input costs [41].

The costs formed were in line with agroindustry research of similar products that use the same calculations, such as research on the financial feasibility analysis of corn-based noodles [42]. Revenue was the income obtained by business actors or the sum of the number of products sold and the price of the products sold so that the components that were taken into account in acceptance consist of the number of products and price [41].

Profit was the difference between total revenue and total costs incurred. Processing agricultural products can increase profits compared to fresh agricultural products that are directly sold [43]. Vermicelli's processing business of corn was one of the efforts to increase profits. The corn vermicelli processing business must be analyzed to assess its feasibility.

Business feasibility analysis needs to be carried out to determine the number of services and expenses made as well as the revenue obtained to analyze the economics of this business activity. Several assumptions are needed. Assumptions are set to assist in data processing, setting the production cost, and making cashflows. The assumptions set include the number of employees' working days, the product's selling price, the increase in expected production capacity, increase in raw material prices, and project life [44].

In business analysis, what we need to know is business capital. Business capital is usually referred to as the initial costs incurred to start a business. According to [45], the cost is a sum of money value issued by producers or entrepreneurs to finance production activities. In business analysis, costs are grouped into fixed and variable costs based on behavior concerning changes in volume.

Fixed costs are production costs that arise due to fixed production factors, even though the number of products produced varies [46]. Meanwhile, variable costs or nonfixed costs are incurred by entrepreneurs as a result of using variable production factors so that these costs vary depending on the number of goods produced in the short term. Costs that include variable costs include direct labor costs and raw material costs [47].

Cost calculations are carried out to include investment costs, semivariable variable costs, fixed costs, and other costs. Investment costs are the amount of capital or costs used to start a business or develop a business [48]. The details of the costs for the analysis of rice flour and corn flour-based vermicelli business can be seen in Table 4.

This is in line with the opinion of [49] that fixed costs are costs that are fixed in amount and continue to be issued even though they occur changes in the volume of production obtained. Fixed costs in the production of vermicelli based on corn and rice flour include electricity and water, depreciation of equipment that supports the production process, and land and building tax. Variable costs are costs incurred when each will carry out the production process. The costs incurred are not fixed depending on the initial capital or income from the previous production. Variable costs include the main and supporting materials needed in one production process.

The price per unit of product based on the total cost is Rp 1,362.00, while the price per unit of product based on variable costs is Rp 1323.00. It can be concluded that this value must be achieved in every sale of one product unit so that a business can be profitable. The cost of production based on the total cost is obtained from the division of the total cost by the final amount of production. At the same time, the price per unit based on variable costs is obtained from the division of variable costs by the final amount of production.

For this business to benefit from each sale of one unit of product, the product per unit sale should be Rp 3,000.00. Table 4 shows that with 110.6 kg of the main ingredient, 1,440 packs of wet vermicelli can be produced, each portion packed with a weight of 100 g, with a total production of 144,000 g.

The selling price that must be achieved to get a profit for each unit of wet vermicelli packaging is Rp 3,000.00 per unit of product to get an income of Rp 4,320,000.00; the amount is obtained from the product of the product unit price multiplication with the number of products produced each time in the production process. So that the net profit can be obtained by selling products per unit is Rp. 3,000.00 is Rp 2,359,150.00. Profit is the difference between the total revenue and the total cost of production.  Table 4 shows that the R/C ratio value of wet vermicelli agro industry based on corn flour and rice flour is 2.27 (based on total variable costs). This value is greater than one, so the wet vermicelli agroindustry based on the mix of corn flour and rice is efficient to run. The R/C ratio value of 2.27 means that every Rp 1.00 issued gets a net profit of Rp 2.27.

## 4. Conclusion

The most affordable wet vermicelli formulation was a mixture of 25% corn flour, 75% rice flour, and 0.6% carrageenan. The yield of wet vermicelli is 144%. The resulting vermicelli has the following characteristics: yellow color, 42.835% moisture content, 0.210%wb ash content, and 0.425%wb fat content. In the organoleptic test, vermicelli that has the criteria of preferred colour (score 3.9), aroma (score 3.6) and soft texture (score 2.6) was moderate. There was a preferred taste (score 3.7) and general acceptance (score 3.5). Economically, making wet vermicelli is profitable with an R/C ratio greater than one, which is 2.27. The resulting wet vermicelli resembles wet noodles, large in size and yellow in color, so it can be recommended as gluten-free noodles, suitable for consumption by people with gluten allergies.

## Figures and Tables

**Table 1:** Physical characteristics of corn and rice grains of several varieties.

No.	Varieties	Seed length (mm)	Seed width (mm)	Seed thickness (mm)	Weight 100 seeds (g)	Classification	Shape
	Corn						
1	DK 88	9.95^b^	8.89^a^	3.85^d^	29.939^b^	—	—
2	Bisi 18	11.43^a^	8.88^a^	4.03^c^	33.694^a^	—	—
3	DK 979	9.90^b^	7.73^b^	4.64^a^	31.017^b^	—	—
4	DK 99	11.07^a^	9.05^a^	4.43^b^	29.493^b^	—	—
5	Pertiwi	11.72^a^	9.47^a^	4.30^b^	34.044^a^	—	—
	Rice						
1	Ciherang	6.7^b^	2.1^a^	—	1.94^bc^	Long	Slim
2	Inpari 19	6.4^c^	2.1^a^	—	1.86^c^	Long	Slim
3	Inpari 4	6.9^ab^	1.9^b^	—	1.88^c^	Long	Slim
4	Inpari 30	6.9^ab^	2.2^a^	—	2.12^a^	Long	Slim
5	IR 64	7.1^a^	2.1^a^	—	2.02^ab^	Long	Slim

Note: the value of a column followed by the same letter is declared not significantly different according to the DMRT test at the 5% level.

**Table 2:** Chemical composition of vermicelli from mixed with corn and rice flour, with the addition of carrageenan.

No.	Formulation		Chemical composition		
	Corn+rice	Carrageenan (%)	Moisture content (%)	Ash content (%wb)	Fat content (%wb)
1	0 + 100	0	30.695^c^	0.160^d-f^	0.355^ef^
2	0 + 100	0.3	40.560^a-c^	0.180^d-f^	0.200^h^
3	0 + 100	0.6	35.100^b^	0.090^ef^	0.340^f^
4	0 + 100	0.9	39.675^a-c^	0.395^a-f^	0.325^f^
5	0 + 100	1.2	40.815^a-c^	0.215^d-f^	0.285^g^
6	25 + 75	0	40.760^a-c^	0.135^d-f^	0.275^g^
7	25 + 75	0.3	42.120^a-c^	0.105^d-f^	0.330^f^
8	*25* + *75*	*0.6*	*42.835* ^a-c^	*0.210* ^d-f^	*0.425* ^a-c^
9	25 + 75	0.9	40.905^a-c^	0.220^d-f^	0.400^b-d^
10	25 + 75	1.2	43.830^a^	0.285^b-f^	0.395^b-d^
11	50 + 50	0	43.220^ab^	0.185^d-f^	0.285^g^
12	50 + 50	0.3	43.830^a^	0.065^f^	0.345^f^
13	50 + 50	0.6	40.980^a-c^	0.205^d-f^	0.385^de^
14	50 + 50	0.9	41.440^a-c^	0.700^ab^	0.415^a-d^
15	50 + 50	1.2	42.030^a-c^	0.220^d-f^	0.450^a^
16	75 + 25	0	39.085^bc^	0.545^a-d^	0.445^a^
17	75 + 25	0.3	41.170^a-c^	0.675^a-c^	0.385^c-e^
18	75 + 25	0.6	40.970^a-c^	0.395^a-f^	0.340^f^
19	75 + 25	0.9	39.900^a-c^	0.535^a-e^	0.415^a-d^
20	75 + 25	1.2	39.630^a-c^	0.725^a^	0.445^a^
21	100 + 0	0	41.740^a-c^	0.115^d-f^	0.410^a-d^
22	100 + 0	0.3	42.025^a-c^	0.255^c-f^	0.230^h^
23	100 + 0	0.6	40.930^a-c^	0.245^d-f^	0.430^ab^
24	100 + 0	0.9	41.215^a-c^	0.235^d-f^	0.445^a^
25	100 + 0	1.2	40.640^a-c^	0.185^d-f^	0.430^ab^

Note: the values in italics (8) are the selected (best) treatment. The value of a column followed by the same letter is declared not significantly different according to the DMRT test at a level of 5%.

**Table 3:** Results of organoleptic test of corn and rice mixture vermicelli.

No.	Formulation (%)		Color	Aroma	Texture	Taste	General reception
	Corn+rice	Carrageenan					
1	0 + 100	0	3.8^a-e^	3.2^a-d^	2.5^c-e^	3.4^a-e^	3.5^a-c^
2	0 + 100	0.3	3.4^c-g^	2.8^b-e^	3.0^a-d^	3.3^a-f^	3.0^b-d^
3	0 + 100	0.6	3.3^e-h^	3.3^a-c^	3.0^a-d^	3.3^a-f^	3.2^a-d^
4	0 + 100	0.9	3.8^a-e^	3.0^a-e^	2.5^c-e^	3.2^a-f^	3.5^a-c^
5	0 + 100	1.2	2.9^f-h^	3.2^a-d^	2.5^c-e^	3.5^a-d^	3.4^a-d^
6	25 + 75	0	3.8^a-e^	3.4^ab^	2.1^e^	3.6^ab^	3.6^a^
7	25 + 75	0.3	4.2^a^	3.4^ab^	2.6^c-e^	3.6^a-c^	3.5^a-c^
8	*25* + *75*	*0.6*	*3.9* ^a-d^	*3.6* ^a^	*2.6* ^c-e^	*3.7* ^a^	*3.5* ^a-c^
9	25 + 75	0.9	4.1^ab^	3.3^a-c^	2.1^e^	3.4^a-e^	3.6^ab^
10	25 + 75	1.2	2.8^h^	2.7^c-e^	2.7^c-e^	2.9^d-f^	2.9^d^
11	50 + 50	0	3.4^d-h^	2.6^de^	2.8^a-d^	2.8^f^	3.0^cd^
12	50 + 50	0.3	3.3^d-h^	2.8^b-e^	2.1^e^	3.1^b-f^	3.2^a-d^
13	50 + 50	0.6	3.7^a-e^	3.3^a-c^	2.8^b-d^	3.0^c-f^	3.1^a-d^
14	50 + 50	0.9	3.5^b-f^	3.1^a-d^	2.9^a-d^	3.1^b-f^	3.2^a-d^
15	50 + 50	1.2	3.4^d-g^	3.2^a-d^	2.5^c-e^	3.1^b-f^	3.1^a-d^
16	75 + 25	0	3.3^d-h^	3.0^a-e^	3.0^a-d^	2.7^f^	3.1^a-d^
17	75 + 25	0.3	3.4^c-g^	3.0^a-e^	3.1^a-c^	2.8^f^	3.2^a-d^
18	75 + 25	0.6	2.8^gh^	2.7^c-e^	2.5^c-e^	3.0^b-f^	3.0^b-d^
19	75 + 25	0.9	3.2^e-h^	3.0^a-e^	2.6^c-e^	2.8^f^	3.1^a-d^
20	75 + 25	1.2	3.5^c-f^	2.7^c-e^	3.3^ab^	3.3^a-f^	3.1^a-d^
21	100 + 0	0	3.3^d-h^	3.0^a-e^	2.4^de^	3.2^a-f^	3.4^a-d^
22	100 + 0	0.3	4.1^a^	3.1^a-d^	3.3^ab^	3.2^a-f^	3.4^a-d^
23	100 + 0	0.6	4.0^a-c^	3.3^ab^	3.4^a^	3.5^a-d^	3.6^a^
24	100 + 0	0.9	3.0^f-h^	3.1^a-e^	2.5^c-e^	2.8^ef^	3.2^a-d^
25	100 + 0	1.2	2.9^f-h^	2.5^e^	2.6^c-e^	2.7^f^	3.1^a-d^

Note: the values in italics (8) are the selected (best) treatment. The value of a column followed by the same letter is declared not significantly different according to the DMRT test at the 5% level. Criteria: color, aroma, taste, and general acceptance: 1 = strongly disliked, 2 = dislike, 3 = moderate, 4 = like, and 5 = really like it. Texture: 1 = very soft, 2 = soft, 3 = moderate, 4 = hard, and 5 = very hard.

**Table 4:** Analysis of production costs for making wet vermicelli based on corn and rice flour mixed.

No.	Cost component/production input	Total	Unit	Price (Rp/unit)	Total (Rp)
I	Total production cost (Rp)				1,960,850
A	Total fixed cost (Rp)				55,750
1	Electricity				30,000
2	Equipment depreciation				25,750
B	Total variable cost (Rp)				1,905,100
a	Main raw material (Rp)				1,050,000
1	Rice flour	75	kg	8,500	637,500
2	Corn flour	25	kg	7,500	187,500
3	Tapioca flour	10	kg	7,500	75,000
4	Carrageenan	600	gr	250	150,000
b	Auxiliary materials (Rp)				855,100
6	Water refill	114	ltr	400	45,600
7	LPG gas contains 12 kg	2.5	tbg	145,000	362,500
8	Packaging plastic	1,440	Sheet	50	72,000
9	Labor	5	Day	75,000	375,000
c	Production				
10	Production (corn vermicelli+rice) (100 g/pack)	1,440	Pack	3,000	4,320,000
11	Cost of production (with total cost)				1,362
12	Cost of production (with variable costs)				1,323
13	Production selling price (set)				3,000
II	Reception	1,440	Pack	3,000	4,320,000
III	Wet vermicelli business advantages				2,359,150
IV	Vermicelli business efficiency (R/C ratio) based on the total cost				2.20
V	Vermicelli business efficiency (R/C ratio) based on variable costs				2.27

Note: the calculation of the production of wet vermicelli per day is 100 kg of flour; the yield of 144% produces 1440 packs/100 g of wet vermicelli.

## Data Availability

Data will be available upon request.
